# Violating the splicing rules: TG dinucleotides function as alternative 3' splice sites in U2-dependent introns

**DOI:** 10.1186/gb-2007-8-8-r154

**Published:** 2007-08-01

**Authors:** Karol Szafranski, Stefanie Schindler, Stefan Taudien, Michael Hiller, Klaus Huse, Niels Jahn, Stefan Schreiber, Rolf Backofen, Matthias Platzer

**Affiliations:** 1 Genome Analysis, Leibniz Institute for Age Research - Fritz Lipmann Institute, Beutenbergstr., 07745 Jena, Germany; 2 Institute of Computer Science, Bioinformatics Group, Albert-Ludwigs-University Freiburg, Georges-Koehler-Allee, 79110 Freiburg, Germany; 3 Institute of Clinical Molecular Biology, Christian Albrechts University Kiel, Schittenhelmstr., 24105 Kiel, Germany

## Abstract

**Background:**

Despite some degeneracy of sequence signals that govern splicing of eukaryotic pre-mRNAs, it is an accepted rule that U2-dependent introns exhibit the 3' terminal dinucleotide AG. Intrigued by anecdotal evidence for functional non-AG 3' splice sites, we carried out a human genome-wide screen.

**Results:**

We identified TG dinucleotides functioning as alternative 3' splice sites in 36 human genes. The TG-derived splice variants were experimentally validated with a success rate of 92%. Interestingly, ratios of alternative splice variants are tissue-specific for several introns. TG splice sites and their flanking intron sequences are substantially conserved between orthologous vertebrate genes, even between human and frog, indicating functional relevance. Remarkably, TG splice sites are exclusively found as alternative 3' splice sites, never as the sole 3' splice site for an intron, and we observed a distance constraint for TG-AG splice site tandems.

**Conclusion:**

Since TGs splice sites are exclusively found as alternative 3' splice sites, the U2 spliceosome apparently accomplishes perfect specificity for 3' AGs at an early splicing step, but may choose 3' TGs during later steps. Given the tiny fraction of TG 3' splice sites compared to the vast amount of non-viable TGs, *cis*-acting sequence signals must significantly contribute to splice site definition. Thus, we consider TG-AG 3' splice site tandems as promising subjects for studies on the mechanisms of 3' splice site selection.

## Abstract 2

TG dinucleotides functioning as alternative 3' splice sites were identified and experimentally verified in 36 human genes.

## Background

Intervening sequences (introns), primary transcript regions that are removed during mRNA maturation, are an outstanding feature of eukaryotic gene structure. Introns are excised through two transesterification reactions involving the collaboration of five different small nuclear ribonucleoprotein particles and additional proteins that associate to form the spliceosome. Rearrangements of the spliceosome and, consequently, splicing catalysis is driven by the sequential action of ATP-dependent helicases [1,2]. The assembly of the early spliceosomal complex relies on sequence-specific contacts between the intron terminal regions and the spliceosome subunits U1, U2, and U2AF [1,3,4]. From accumulating intron sequence data, it was noted that invariant dinucleotides must represent important signals for the definition of intron termini, the so-called GT-AG rule (for simplicity, we use the nucleotide symbol T to denote thymidine in DNA as well as uridine in RNA sequences). With respect to the role of intron termini in the transesterification reactions, the 5' GT site and the 3' AG site were named donor and acceptor splice sites, respectively.

Early work on unusual splice signals revealed introns with the terminal dinucleotides AT-AC [5], and these were later shown to be processed by an independent splicing pathway, the U12 spliceosome. The U12 spliceosome recognizes highly specific donor site and branchpoint motifs [6] while recognition of 3' splice sites is rather unspecific. As a result, there are several variants of intron termini besides the prominent combinations GT-AG and AT-AC [7,8].

Among U2-dependent introns, the most frequent exception to the GT-AG rule are GC-AG intron termini, which comprise 0.7-0.9% of vertebrate introns [5,8,9]. Other rare exceptions are GA-AG intron termini in the FGFR gene family [10] and AT-AC termini, mostly found in introns of the SCN gene family [8,11,12]. While the latter cases are the only reported non-AG 3' splice sites, results from *in silico *studies have repeatedly suggested that other unusual 3' splice sites occur in U2-type introns [13,14]. An in-depth, systematic screening effort could not reveal significant evidence for additional unusual intron 3' termini above the noise level brought by annotation errors [9]. However, it was noted that a few exceptional U2-spliceosomal introns exist that involve unusual 3' splice sites in scenarios of alternative splice site choice. For example, intron 3 of the human guanine nucleotide binding protein gene *GNAS *is spliced at either TG or AG in the 3' intron sequence CTGCAG [15,16]. Remarkably, the homologous *Drosophila *gene shows the same unusual splicing pattern for another intron [17]. Moreover, unusual TG splice acceptors appear to be involved in alternative splicing of the human gene for presynaptic density protein 95, *DLG4 *[18], and the human dopamine D2 receptor gene, *DRD2 *[19].

We have previously reported a widespread type of alternative splicing mediated by the tandem splice acceptor motif NAGNAG [20]. From the analysis of single-nucleotide polymorphisms (SNPs) we concluded that a NAGNAG motif is necessary and sufficient to explain three-nucleotide variant splicing at intron-exon boundaries [21]. In contrast, alternative splicing of an intron 3' terminus in the *GNAS *gene appears to occur independently of a NAGNAG motif. Furthermore, it has been suggested that unusual splice sites could be selectively involved in alternative splicing [5,9], although this was never examined in detail. Here, we report a systematic screening of the human transcriptome that identified 36 introns with *bona fide *TG 3' splice sites. These TG splice sites are exclusively found as alternative 3' splice sites, each associated with a canonical AG 3' splice site. The evolutionary conservation of these introns and their alternative splicing patterns indicate physiological relevance and point to the requirement for *cis*-regulatory sequence elements to promote usage of TG 3' splice sites.

## Results

### Prior considerations

We used an *in silico *approach based on expressed sequence tags (ESTs) to identify unusual 3' splice sites that are found in pairs of 3' splice variants. ESTs, as first-pass results from high-throughput cDNA sequencing projects, are clearly prone to errors. Therefore, we assumed that single ESTs are insufficient to indicate genuine subtle splice variants since technical artifacts contribute to false positives. We considered a variant as sufficiently evident if it is supported by at least two independent ESTs, and expect the EST variant ratio to serve as an approximation for the natural ratio of splice variants. An additional threshold was applied to the relative abundance of splice variants, since our experimental approach, that is, sequencing of 100 individual RT-PCR clones, had a detection limit. Using a random binomial distribution to model the occurrence of splice variants in the RT-PCR clones, we calculated a diagnostic power of 95% (β error 5%) if a splice variant occurs with at least 3% frequency. It is important to note that we have not inferred anything for the cases that failed the threshold criteria. It is possible that they actually represent natural splice variants; however, the evidence for such cases is weak and the experimental approach did not provide sufficient sensitivity for validation.

### TG dinucleotides function as non-canonical alternative 3' splice sites

**Figure 1:**
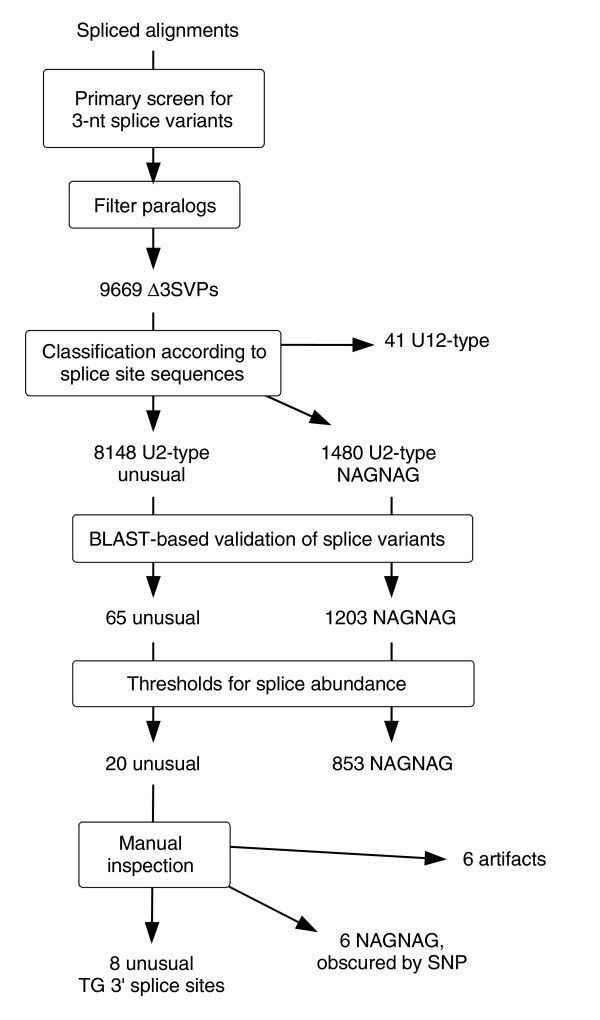
Screening procedure for unusual 3' splice sites found in pairs of 3' splice variants that differ by 3 nt (Δ3SVPs). Processing of AG-AG tandem cases ('NAGNAG', parallel branch on the right) was performed as a comparison to unusual 3' splice site tandems.

We initially aimed to identify unusual 3' splice sites that are found in pairs of 3' splice variants that differ by 3 nucleotides (nt), such as in the *GNAS *intron 3 splice site tandem [15,16]. Identification of 3 nt splice variant pairs (Δ3SVPs) was based on 3' splice sites as indicated by spliced alignments of human ESTs [22]. After a reduction of false positives performed by a series of filtering steps (Figure 1), we identified 65 'unusual' Δ3SVPs that were supported by high-quality local EST alignments. Of these, 20 meet the requirements that the minor splice variant is supported by at least two ESTs and 3% of the matching ESTs (see considerations below). However, after close inspection and re-sequencing, we identified 6 of the 20 unusual Δ3SVPs as false positives (Additional data file 1), explained by: 3 nt deletion variants due to sequencing errors; mouse ESTs erroneously attributed to human; or alignment artifacts. Another six Δ3SVPs can be explained by SNPs, where the SNP allele corresponding to a NAGNAG splice site motif is not displayed by the human reference genome sequence [21]. Strikingly, all the remaining eight Δ3SVPs suggest that TG dinucleotides function as alternative 3' splice sites (Table 1).

**Table 1:** Unusual TG splice acceptors identified in the human transcriptome

	Intron		3' Splice site pair		ESTs for unusual 3' splice sites	
			
Gene	No.	Length	Distance	Motif	Fraction	No.
*GNAS*	3	7843	3	CTG,CAG|	0.15-0.62*^†^	282
*PCGF2*	1	224	3	AAG|ATG,	0.50	4
*CNBP*	3	168	3	TTGTTG,AAG|	0.25	257
	3	168	6	TTG,TTGAAG|	0.01	10
*FBXO17*^‡^	3	1999	3	CAG|ATG,	0.14	4
*C21orf63*	3	9975	3	CAG|ATG,	0.09	2
*BRUNOL4*	6	1147	3	CAG|CTG,	0.07	2
*PCID2*	2	2162	3	CAG|ATG,	0.04	7
*TNNT2*	1	4354	3	TTG,GAG|	0.04	2
***CACNA1A***	9	2532	3	TTGTTG,GAG|	?^†^	-
	9	2532	6	TTG,TTGGAG|	0.17^†^	-
*GPBP1*	9	1377	4	CAG|GATG,	0.03	2
*KIAA0494*	1	1459	5	TTG,AGCAG|	0.09	2
*OSBPL8*	2	36530	6	CTG,TTGTAG|	0.11	2
*SAP30*	1	1892	6	CTG,TTTCAG|	0.04	2
***DRD2***	6	1485	6	CTG,GTGCAG|	0.02^†^	5^†^
*SUV420H2*	5	134	7	CTG,GCTCCAG|	0.20	3
*SSRP1*	1	489	7	TTG,AATTCAG|	0.20	16
*FREQ*	2	16849	7	CTG,CCTCCAG|	0.04	2
*IL21*	3	2753	8	TTG,ATTTCTAG|	0.13	2
***RYK***	7	3107	9	TTG,GCTCCTTAG|	0.77	27
***DLG4***	5	131	9	CTG,GAGTTGCAG|	0.62	8
*SMARCA4*	29	6174	9	TTG,ACCCTGAAG|	0.41	34
*FBXL10*	15	177	9	TTG,GCCTACAAG|	0.21	3
*HNRPR*	7	2839	9	TTG,GTTTAACAG|	0.13	15
*RRAD*	1	214	9	CTG,ATCCCCTAG|	0.06	2
*TGM1*	6	454	10	CTG,TCCTGGGCAG|	0.13	2
*ALAS1*	11	1599	11	CTG,TTTCTCCTCAG|	0.04	5
***ARS2***	18	182	12	TTG,TACTCCCCCCAG|	0.74	75
***PCBP2***	7	1337	12	CTG,ACTCTCTCCCAG|	0.43	169
*PTPN11*	10	4269	12	TTG,GCTCTACTCCAG|	0.33	3
*MSH5*	6	164	12	CTG,ATCCCCTCCCAG|	0.25	5
*SYTL2*	9	1259	13	TTG,CCCTCCTGAGTAG|	0.09	3
*TOMM40*	1	95	16	CTG,ACCTCTCCCCTAGCAG|	0.07	2
*MARK3*	3	20478	17	TTG,TTTGTTTTTTTTTTTAG|	0.07	3
***BAT3***	6	832	18	CTG,ACTCTCCCCTACCTTCAG|	0.01	1
***SH3D19***	6	838	21	TTG,GTTTTGTTTTGGTCTCGTCAG|	0.07	1
***LOC346653***	1	3097	27	CTG,ACCCATGTACCTGAGGCTGATTTCCAG|	0.60	3
*ACAD9*	10	253	28	TTG,TTTCTTGTGTTTTTTCTGAACACTCCAG|	0.09	4

Entries in bold have RefSeq transcripts supporting the unusual TG acceptor site. Each TG splice variant is supported by at least two ESTs and at least 3% of all covering ESTs, except for some RefSeq-supported cases, *CACNA1A *[24,35]*, DRD2 *[19] and *BAT3*. In the 'Motif' column, a vertical line (|) indicates a canonical splice site, and a comma (,) marks the TG splice site. Splice ratios are given as absolute EST counts (No.) as well as the fraction of TG splice variants. A question mark indicates that an explicit fraction is not given in the referenced article, although the authors performed quantitative experiments. *EST ratio depends on the exon junction; the upstream exon 3 may be skipped. ^†^Splice variants were previously quantified by others: *GNAS *[16,26], *CACNA1A *(splice ratio cited from [24,35]), *DRD2 *(splice ratio cited from [19]). ^‡^Alternative splicing at *FBXO17 *intron 3 was not experimentally reproducible in this study.

Since all the unusual alternative Δ3-nt splice acceptors identified display TG dinucleotides, we investigated their occurrence in a wider scope. Analogously to the screen for Δ3SVPs (Figure 1), we performed a search for alternative TG splice acceptors at larger distances, up to 36 nt from from the canonical splice site. The same filter procedures were applied, and close inspection did not reveal obvious artifacts or explanatory SNP alleles. We identified 26 additional EST-supported splice variant pairs that suggest alternative TG splice acceptors functioning in U2-dependent introns (Table 1).

We sought to screen for unusual splice acceptors using an independent approach in order to cross-validate our initial findings and to make a link to previous studies that were primarily based on curated transcript data [5,9]. An analysis of RefSeq-to-genome alignments identified 122 putative introns with terminal TG dinucleotides (120 unique genomic sites) out of 228,925 total introns (171,605 unique genomic sites). Of these, 39 introns have a canonical GT donor dinucleotide (Additional data file 1). A previous study, performing a similar screening approach for unusual splice sites using curated transcript data, showed high enrichment of annotation artifacts [9]. Therefore, we checked the identified TG acceptor cases thoroughly. In fact, cases failed this quality check for several reasons: known SNPs masking existing canonical AG splice sites; RefSeqs lacking transcript evidence; and misleading RefSeq-to-genome alignments. Since the overall false positive rate seemed very high, we additionally required independent transcript entries (mRNA or EST) to support the unusual splice site. In summary, 9 of the 39 RefSeqs showed robust support for unusual TG acceptor sites (Table 1, entries in bold), and 6 of these overlap with cases obtained from the EST screening approach while the others are exclusively identified by the RefSeq-based approach (*SH3D19*, *BAT3*, *CACNA1A*). Intriguingly, these three EST-independent RefSeq-supported cases all comprise 'alternative' splice sites, although this was not a screening criterion. Taking into account that about 1% of all introns have alternative 3' termini [23], this strongly indicates that TG splice acceptors are functionally linked to nearby AG splice sites and cannot function in a constitutive manner (*P *= 0.000001, binomial test). Altogether, the two screens identified 37 introns with 39 alternative TG splice acceptors (Table 1).

### Negation of genome sequence errors and polymorphisms

Since six putatively unusual 3' splice sites can be explained by SNP-affected NAGNAG acceptors (which were filtered; Figure 1), we asked whether undiscovered SNPs, or even inaccuracies in the available human genome sequence, may explain some of the remaining candidates. The genomic sequence of the splice site regions of *GNAS *and *CACNA1A *had been experimentally verified by others [16,24]. For 10 other genes (listed in Table 2), we analyzed PCR products obtained from genomic DNA, pooled from 100 individuals, for sequence variations. The re-sequenced genomic regions were in perfect agreement with the available genome sequence, negating the possibility that unusual splice sites are trivial sequencing errors (data not shown). Moreover, we identified no SNP alleles that confer explanatory AG splice sites on any of the observed unusual splice variants, demonstrating that the TG splice sites are real and genetically invariant.

**Table 2:** Validation and quantification of alternative splice variants

Splice junction		Tissue	Fraction of TG splice	Method
*GNAS*	Intron 3, exon junction 3-4	Leukocytes	0.14	n = 115
*PCGF2*	Intron 1	Placenta	0.99	n = 89
*CNBP*	Intron 3, indel AAG	Leukocytes	0.52	n = 69
		Brain	0.38	n = 58
		Placenta	0.33	n = 70
	Intron 3, indel TTGAAG	Leukocytes	0.01	n = 69
*FBXO17*	Intron 3	Leukocytes	-	n = 96
		Liver	-	n = 151
*C21orf63*	Intron 3	Leukocytes	0.09	n = 110
		Brain	-	Direct sequencing
*BRUNOL4*	Intron 6	Lung	-	n = 90
		Brain	0.19	n = 92
*PCID2*	Intron 2	Leukocytes	0.02	n = 142
*TNNT2*	Intron 1	Heart	0.03	n = 91
*CACNA1A*	Intron 9, indel GAG	Brain	0.85	n = 90
	Intron 9, indel TTGGAG	Brain	0.03	n = 90
*ARS2*	Intron 18	Leukocytes	0.55	n = 37
*PCBP2*	Intron 7	Leukocytes	0.54	n = 47
*LOC346653*	Intron 1	Testis	0.50	Direct sequencing

In the 'Methods' column, n represents the number of subclones sequenced.

### Validation and quantification of splice variants

To verify the existence of TG-derived splice variants, we performed RT-PCR experiments designed to yield amplicons that cover the exon-exon junctions under consideration. Cloning of the PCR products and sequencing allowed us to detect splice variants. Subclassification and counting of clones gave measures of splice variant ratios. This way, the alternative splicing pattern was reproduced and quantified for 11 out of 12 analyzed cases (Table 2). Generally, the splice ratios obtained from clone counting agree well with the EST data. The observed deviations can be explained by significant fluctuations depending on the analyzed tissue (*C21orf63*, *BRUNOL4*, and *CNBP *in Table 2). The splice variant validation failed for *FBXO17*, a gene for which 4 out of 29 ESTs had suggested a TG-derived splice variant. All supporting ESTs originated from the same EST library, NIH_MGC_100, derived from a hepatocellular carcinoma. A peculiarity of the source material, either the NIH_MGC_100 cell line or the single-individual liver sample used for our RT-PCR experiments, may be the reason for the inconsistent results concerning this putative splice variant. This example illustrates that at least two ESTs from independent sources are required to indicate a natural splice variant with high reliability. Overall, the success rate of the validation experiments was high (92%), and extrapolated to the 25 non-tested cases, about 2 false positives are expected.

### Tissue-specificity of splice ratios

**Figure 2:**
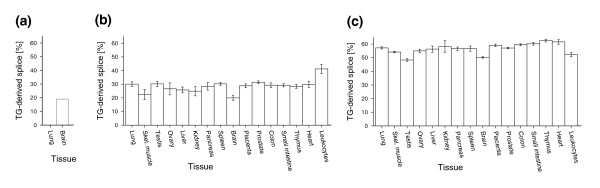
Tissue-specific fractions of TG-derived splice variants. **(a) ***BRUNOL4 *(values are as shown in Table 2); **(b) ***CNBP*; and **(c) ***ARS2*. Pyrosequencing assays (for (b,c)) were performed multiple times for each sample (two to four times). Error bars depict the standard deviation of individual measurements.

According to the results of the PCR cloning approach, *BRUNOL4 *displayed remarkable tissue-specific splice ratios. The TG-derived splice variant was not detected in lung cDNA whereas the same variant constituted 20% of brain *BRUNOL4 *transcripts (Table 2, Figure 2a). So we asked if splice ratios of TG-derived and AG-derived variants generally show tissue-specific differences. We analyzed the splice variant ratios more extensively in other genes using pyrosequencing, a method that allows accurate and cost-effective quantification of mixtures of polymorphic DNA populations [25,26]. *ARS2 *and *CNBP*, both having a ubiquitous expression profile, show tissue-dependent fluctuations in splice ratios (55-65% and 20-40%, respectively; Figure 2). While these differences are numerically significant in each of these genes (α = 0.01, ANOVA), their biological relevance is debatable. We conclude that splice ratios of TG-AG tandems are tissue-specific for particular introns but are rather stable for others.

### Evolutionary conservation of introns with TG 3' splice sites

**Figure 3:**
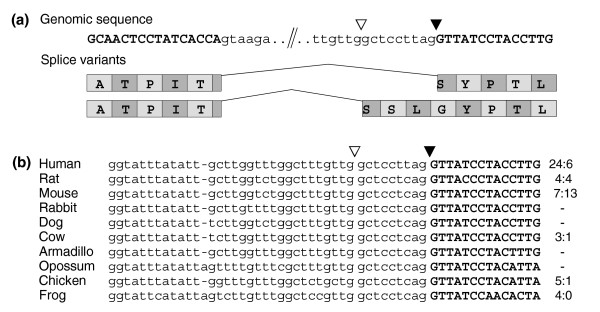
Conservation of the TG splice site found in intron7 of the *RYK *gene from human to chicken. **(a) **Human genomic sequence and derived splice variants. Canonical (filled triangle) and TG 3' splice site (open triangle) are marked. **(b) **Alignment of orthologous exon-intron boundary regions from several vertebrate genomes, splice sites highlighted as in (a). Numbers on the right display the ratios of species-specific ESTs for the TG and AG splice sites, respectively.

Since splicing at TG sites occurs in a very small number of introns, one might argue that these represent 'accidental' events attributable to spliceosome dysfunction. To address this question, we first analyzed the conservation of splice sites in homologous introns as an indication of alternative splice variants being under purifying selection. Out of 36 introns with 3' TG splice sites (37 minus the false-positive *FBXO17 *intron), 26 (72%) are conserved between the human and mouse genomes. In 14 of these cases (39% of the total), mouse ESTs indicate homologous TG-derived splice variants. For comparison, this rate is three- to four-fold higher than that of alternative exons found in both human and mouse [27-29]. In some cases, EST evidence for orthologous TG-derived splice variants even exists for distantly related species, such as chicken (*CNBP*, *BRUNOL4*, *RYK*), and frog or fish (*BRUNOL4*, *RYK*, *FBXL10*). An outstanding example of conserved intron sequence and homologous splice variants is intron7 of the *RYK *gene (Figure 3). The ratio of 3' splice variants is remarkably similar between human and chicken, as can be inferred from the available EST data (EST ratios of 24:6 and 5:1 for human and chicken, respectively). In general, it should be noted that homologous splice variants may remain undetected due to the limited depth of EST coverage [23,27].

**Figure 4:**
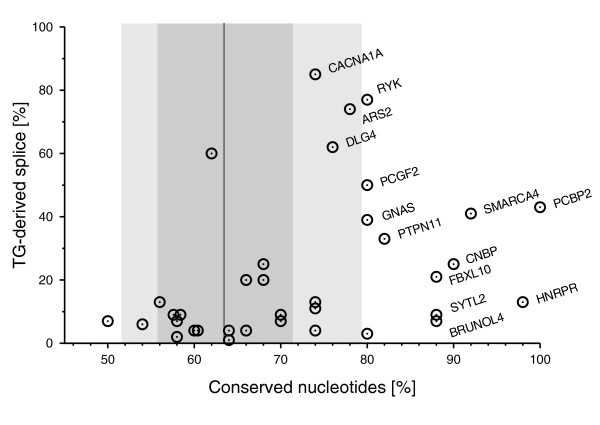
Intron flank conservation of TG-AG splice acceptor tandems. Orthologous human/mouse intron-exon boundaries involving TG splice sites are displayed in a two-dimensional plot according to two properties: horizontal axis = sequence identity of 50 nt sequence upstream of both splice sites; vertical axis = relative abundance of the TG-derived splice variant, as reflected by the fraction of TG-spliced ESTs (except for *CACN1A1*, where the data are taken from Table 2). Data points are labeled with the gene symbol if the conservation score and/or the fraction of TG-derived splice variant are significantly high. Conservation properties of canonical introns are indicated by shaded intervals: black line = median; dark gray = 66% percentile; light gray = 90% percentile.

Independently, we analyzed intron sequence conservation as an indication of the functional relevance of alternative splicing [27-29]. A data set of human-mouse orthologous intron-exon boundaries was used to determine the degree of conservation within a 50 nt intron sequence upstream of the splice acceptor, or acceptor tandem. Intronic flanks of TG splice sites show an average sequence similarity of 74%, whereas flanks of AG splice sites within canonical (AG-only) introns are 65% similar on average (*P *<< 0.00001, permutation test). A plot of flanking sequence conservation against the abundance of the TG-derived splice variant (Figure 4) shows that these two measures are positively correlated. Introns that give rise to less than 10% TG-derived splice variants have an average human-mouse intron sequence identity of 64%, indistinguishable from canonical introns. In contrast, introns with a TG-derived splice variant making up more than 10% of the transcripts show an average sequence identity as high as 80%. This parallels a previous finding that the abundance of splice variants correlates with sequence conservation of alternative exons [27]. Consistently, high intron sequence conservation is strongly correlated with conservation of the TG splice site (13 of the 14 cases with gene labels in Figure 4). Our results indicate that splicing at TG acceptors may arise from neutral evolution, presumably showing low splicing efficiency. However, efficiently spliced TG 3' splice sites seem to evolve and to be maintained by evolutionary selection.

### Structural and sequence characteristics of TG 3' splice sites

We analyzed the context properties of TG 3' splice sites in order to find an explanation for these rare exceptions to the GT-AG rule. With regard to the gene structure context, TG-splicing introns are indistinguishable from canonical introns in several respects: length of the affected as well as the downstream introns, and length of the upstream and downstream exons (data not shown). TG 3' splice sites are significantly often found in the first intron of the gene; 8 of 36 (22%) TG-AG introns compared to 11% of other introns (*P *= 0.02, Fisher's Exact). This bias is also found for AG-AG splice site tandems and is certainly due to neutral evolution of introns located in the 5' untranslated transcript region.

TG 3' splice sites were exclusively found within a context of alternative splice site choice (Table 1). This is clearly significant for results from the RefSeq-based screening procedure, which are unbiased with respect to constitutive or alternative transcripts. Taking into account that about 1% of all introns have alternative 3' termini [23], it strongly indicates that TG 3' splice sites are functionally linked to nearby AG splice sites and cannot function in a constitutive manner. This conclusion is further supported by studies of human AG>TG 3' splice site mutants [30-32], which always resulted in activation of neighboring AG splice sites, but not splicing at TG. Furthermore, we observed that TG-AG tandems display a splice site distance restriction with a limit of 28 nt, which is not seen for AG-AG tandems (Table 1, Additional data file 1). Thus, splicing of a 3' TG does not depend only on an additional AG splice site, this dependency also seems to pose a constraint on TG-AG splice site distance. The observed distance limit corresponds well with the distance between the branch site and the 3' splice site, which is typically 20-40 nt [33].

Splice site strength was scored using the maximum entropy method 'maxent' of Yeo and Burge [34]. The 5' splice site scores are indistinguishable between TG-AG introns and canonical introns. On the other hand, the AG 3' splice sites in TG-AG tandems score significantly lower than canonical introns (6.1 ± 3.5 versus 8.5 ± 2.8, respectively; *P *= 0.00001, Student's *t*-test). The 3' splice site score remains significantly small if low-scoring outliers are excluded from the analysis (6.7). Interestingly, the sequence context of TG 3' splice sites is very similar to canonical AG splice sites in that it shows a preference for pyrimidines at position -3 and preference for purines at position +1 (Additional data file 1). However, TG 3' splice sites changed to AG yield an average score of 6.6, again significantly lower than that of canonical AG splice sites (*P *< 0.00001, Student's *t*-test). This disfavors the simple explanation that TG and AG splice sites compete for the same recognizing factors and the neighboring nucleotide composition (that is, the feature scored by maxent) alone acts to direct splice site choice towards TGs. Finally, it remains questionable if maxent, trained on canonical AG 3' splice sites, has any predictive power for the functionality of TG splice sites.

The fraction of TGs functioning as splice acceptors is extremely small, about 0.01% of candidate motifs. Thus, *cis*-regulatory elements must play a crucial role in the definition of TG splice acceptors. From the ratio of functional/non-functional TG-AG tandems, in comparison with AG-AG tandems, we estimate that at least 6 nt of *cis*-regulatory sequence information is required to promote splice site usage of 3' TGs (Additional data file 1). This is in agreement with 5-20 unchanged nucleotides in excess over the average intron mutation rate, found in about half of the TG-splicing introns, that is, those considered to be subject to purifying selection for splicing of 3' TGs. However, we failed to identify specific regulatory motifs (data not shown). This may be due to the dispersed arrangement of *cis*-regulatory elements, or the contextual cooperation of diverse elements. Due to the relatively small sample size for TG 3' splice sites, available methods for motif discovery have limited detection power.

## Discussion

Previous studies provided incidental evidence for unusual 3' terminal dinucleotides in U2-dependent introns, particularly TG dinucleotides that are used as alternative 3' splice sites. Few directed efforts have been made so far to verify such instances and to elucidate underlying mechanisms and consequences [16,35]. Here, we report 36 human U2-spliceosomal introns with TG dinucleotides functioning as 3' splice sites, identified by thoroughly filtered EST-to-genome alignments. The high accuracy of the EST-based screening approach was validated by RT-PCR with a success rate of 92%. Though it might seem paradoxical, the analysis of EST data gave superior results compared to an analysis of curated data, that is, RefSeq transcripts. We found that the abundance of EST data allows the application of statistical methods for obtaining valid results whereas curated data sets, which are typically devoid of redundancy, may contain errors that are rarely captured by filtering criteria, consistent with the findings of others [36]. In practice, we found that two independent ESTs are strong evidence for a natural splice variant. Given this rather permissive threshold [9,37], we expect that the established screening protocol achieves high sensitivity.

Since our screening procedure is EST-based, certainly more unusual 3' splice sites remain undiscovered in transcript regions that lack sufficient EST coverage. Moreover, there are indications that even other unusual dinucleotides, apart from TG, may function as alternative 3' splice sites. For example, others reported an AT 3' splice site in the mammalian *DGCR2 *gene [8], a CG 3' splice site in the *Drosophila per *gene [38], and we found that a TG splice acceptor in human *CNBP *intron 3 is replaced by a viable GG in the chicken ortholog (results not shown). The occurrence of a TG splice acceptor in the *Drosophila gnas *gene suggests that they occur throughout metazoan organisms.

Other studies have questioned the extent to which alternative splicing is functionally relevant [27-29]. Since TG splice acceptors are extremely rare compared to AG acceptors, one might think that these cases reflect a fuzziness of the splicing reaction. However, multiple findings support the idea that TG splice sites are activated by directed mechanisms and that the resulting splice variants fulfill functional roles: first, several TG splice acceptors are used with a high frequency or can even be the preferred splice site, which excludes splicing errors as a plausible explanation (Table 1, Figure 4); second, TG splice acceptors and their adjacent intron sequence are remarkably conserved between orthologous mammalian genes (Figure 4); third, tissue-specific splice patterns are observed for *GNAS *[16,26] as well as *BRUNOL4 *(this study; Figure 2), suggestive of specific regulatory processes; and fourth, the TG splice site-mediated protein isoform of the mammalian calcium channel subunit α_1A _(*CACNA1A*) has been shown to result in significant differences in neuronal excitability [35].

Thinking of splice site evolution as a process of functional engineering, we might ask about the functional options that distinguish TG-AG splice acceptor tandems from AG-AG tandems. During analysis of orthologs of human TG splice acceptors, we did not identify any case of orthologous AG splice sites, suggesting that TG and AG splice site dinucleotides are functionally non-equivalent. The inserted/deleted nucleotide sequence differs only if TG is positioned downstream of the tandem splice site. Apart from the possible impact on the protein sequence, an NAGATG tandem acceptor allows insertion of a start codon. For example, this seems to be realized in intron 1 of human *PCGF2*, where the observed splice variants differ by the presence of an upstream open reading frame. Preliminary results indicate that this ATG insertion has an effect on the translation efficiency of the mRNA (results not shown). It is also worth noting that the *Drosophila gnas *gene has a TG splice acceptor, like the human gene, but it is located in a non-homologous intron [17]. Given the overall low frequency of TG 3' splice sites (0.02%), this example of convergent evolution indicates a functional benefit of the unusual splice site, independent of its impact on protein sequence. It is tempting to speculate that splicing of TG splice acceptors, rather than providing a pathway for alternative transcripts or protein isoforms, may play a role as a regulatory bottleneck for maturation of the transcript, as was suggested for U12-type introns [39].

Considering functional classes, a significant fraction of TG-spliced genes represent regulators of chromatin structure (*PCGF2*, *GPBP1*, *SAP30*, *SUV420H2*, *SSRP1*, *SMARCA4*) as well as splicing factors and translational modulators (*CNBP*, *BRUNOL4*, *HNRPR*, *PCBP2*). Interestingly, two of the affected RNA-binding proteins are reported to bind DNA as well [40,41]. Together, these enrichments suggest a regulatory cross-talk between transcription on the one hand, and splicing, mRNA maintenance, and translation on the other. Together with another subgroup associated with receptor-mediated signal transduction (*GNAS*, *DRD2*, *FREQ*, *IL21*, *RYK*, *DLG4*, *RRAD*, *PTPN11*, *SYTL2*, *MARK3*, *SH3D19*), most of the genes' functions may be circumscribed with 'information processing', a term that was introduced to describe the functional characteristics of U12-dependent introns [6]. However, as a statistical analysis of Gene Ontology functional classification terms does not reveal any significant over- or under-representation (results not shown), further work is required to determine the relevance of these findings.

TG-AG splice acceptor tandems illustrate the flexibility as well as the specificity of splice site selection by the U2-type spliceosome. The spliceosome is flexible enough to choose TG dinucleotides as splice acceptors. Despite this flexibility, a TG splice site depends on a neighboring AG splice acceptor, since constitutive TG splice acceptors are not found, and TG-AG acceptor tandems show a distance constraint. We assume that an AG splice acceptor, within the typical context of a branch-point motif and polypyrimidine tract, is essentially required for intron definition to promote splicing stepI *in vivo*. Consistent with this, a recent report showed that the essential splicing factor U2AF^35 ^in cooperation with other factors mediates the spliceosome's specificity for AG 3' intron termini during splicing stepI [42]. Assuming that splicing stepI does not ultimately define the 3' splice site, we hypothesize that definite splice site choice takes place during reaction stepII, allowing TG dinucleotides to function as 3' splice sites. Since U2AF dissociates from the spliceosomal complex after stepI [43,44], other factors may influence splice site choice at a later step. Two different modes of 3' splice site selection after splicing stepI have been suggested for AG-AG splice site tandems. First, a second 3' AG may be chosen as the site of exon ligation during splicing stepII if it is located a few nucleotides downstream of the first-step AG, defined by U2AF binding [45]. This rather unspecific mechanism is the likely explanation for the high propensity of small-distance AG-AG tandems to result in alternative splicing, and may also be relevant for TG-AG acceptor tandems, which are found overrepresented at a 3-nt distance compared to larger distances (Figure S1 in Additional data file 1). Another mechanism is exemplified by intron 2 of the *Drosophila sxl *gene [46] as well as intron 1 of the β-globin mutant β^110 ^[47,48]. Here, the downstream AG is essential for splicing while the dispensable upstream AG may be chosen in splicing stepII, even as the preferred splice site. The splicing factor SPF45 was shown to bind to the upstream AG dinucleotide during splicing stepII, promoting splice site choice [46]. It remains to be tested if SPF45 or other factors contribute to TG splice site choice.

Given the extremely low ratio of viable versus non-viable TG-AG tandems at intron-exon boundaries, contextual sequence signals must contribute to TG splice site definition and influence splice site choice. In agreement, half of the TG splice acceptors are associated with outstandingly high intron sequence conservation. Notably, the alternative TG splice acceptor of *GNAS *intron 3 has been shown to be flanked by three putative exonic splice enhancer motifs (specific for SF2/ASF, SC35, and SRp40), and TG splice site choice has been experimentally shown to be modulated by the ratios of SF2/ASF and hnRNPA1 [16]. We could not identify specific sequence motifs associated with TG splice sites (results not shown). Due to the relatively small sample size for TG 3' splice sites, available methods for motif discovery have limited detection power, especially if *cis*-regulatory elements are highly dispersed, or if diverse elements cooperate in a contextual manner. Presumably, each individual TG-AG tandem recruits a characteristic ensemble of splice regulators to facilitate unusual splice site choice. Thus, the compilation of TG splice sites could serve as a rich source of splicing-relevant contextual sequence signals to be examined in future experimental studies.

## Materials and methods

### Screening for non-canonical 3' splice sites

From the UCSC Genome Browser site [22] we obtained spliced alignments of human ESTs (file all_est.txt, released 2005-07-14) and of human RefSeq transcripts (refGene.txt, 2005-07-23) [49], as well as a compilation of all human EST sequences (est.fa, 2005-11-26). First, we sampled EST-supported 3' splice sites to identify 3-nt splice variant pairs (Δ3SVPs). In parallel, we identified ESTs that were mapped to multiple genome locations, indicative of paralogous gene loci including pseudogenes. We discarded those Δ3SVPs whose EST support for the minor splice variant did not exceed the number of these ambiguously mapped ESTs. Furthermore, we retained only those Δ3SVPs that have at least one splice site corresponding to a RefSeq transcript, according to the RefSeq-to-genome alignment. Then, we separated cases that involve the dinucleotide AG at both 3' splice sites, that is, NAGNAG tandem splice acceptors, as well as U12-dependent introns, identified by their characteristic donor site and branch-point motifs [6]. The remaining Δ3SVPs were considered 'unusual' since they comprise at least one non-AG splice acceptor in a U2-spliceosomal intron. The splice variants of these Δ3SVPs were validated and quantified by a WU-BLASTN search of 60-nt sequence windows around the resulting exon-exon junctions against all human ESTs, using parameters W = 13, N = -8, nogap S = 180, hspmax = 1. BLAST matches were considered valid if perfect sequence identity was found in a 12-nt window around the exon-exon junctions [37]. Finally, Δ3SVPs were considered highly reliable if the minor 3' splice site was found in at least two ESTs and was used in at least 3% of the covering ESTs. A screen for splice variant pairs for distances of 4-36 nt was performed analogously, restricting the search to tandems of TG-AG splice sites.

### PCR and RT-PCR

For validation of splice variants, nested PCR was performed using 1 ng cDNA templates from the Human Multiple Tissue cDNA PanelsI and II (Clontech, Mountain View, CA, USA). For a given gene, suitable tissues were determined from expression data obtained from the Stanford SOURCE database [50]. However, pooled leukocyte cDNA from 200 individuals was preferably chosen in order to obtain comparable results. Verification of the genomic sequence and an analysis of potential polymorphisms were done by nested PCR using 200 ng of pooled genomic DNA from 100 Caucasian individuals (Roche, Mannheim, Germany) as template. Primers were obtained from Metabion (Martinsried, Germany) (Additional data file 1). Reactions were set up with PuReTaq Ready-To-Go PCR beads (GE Healthcare, Munich, Germany) and 10 pmol primer in 25 μl total volume, according to the manufacturer's instructions. A typical thermocycle protocol was 3 minutes initial denaturation at 94°C, followed by 25 cycles of 1 minute denaturation at 94°C, 1 minute annealing at 53-55°C, 1 minute extension at 72°C, and a final 10 minute extension step at 72°C. In the second round of nested PCR, 1 μl of the first-round product was amplified for 30 cycles. For cloning, PCR products were separated on agarose, DNA was extracted applying the Millipore (Billerica, MA, USA) Montage Gel Extraction kit, followed by ethanol precipitation. Isolated fragments were cloned in pCR2.1-TOPO (Invitrogen, Karlsruhe, Germany), and cloned DNA was Sanger sequenced using M13 standard reverse primer (17-mer).

### Splice variant quantification by pyrosequencing

Templates for pyrosequencing were generated using universal biotinylated primers [51]. RT-PCR amplicons of the exon-exon junctions were ligated into pCR2.1-TOPO (Invitrogen) according to the supplier's recommendations and subsequently re-amplified with all four possible combinations of 5'-biotinylated M13 standard primers (17-mers) and unlabeled insert-specific primers (Additional data file 1). The latter also served to prime the pyrosequencing reaction. Biotin-labeling of DNA, single strand preparation and sequencing were performed as described [51].

### Orthologous intron-exon boundaries

A data set of orthologous intron-exon boundaries was constructed automatically to obtain sufficient data (especially reference data) to test for evolutionary constraints on intron flanking sequence. Sets of human (data as described for the splice site screen) and mouse transcript annotations (UCSC genome assembly mm7, RefSeq-to-genome alignment 2006-05-21) were processed as described earlier (supplementary methods in [20]). For 97,107 unambiguous orthologous pairs (57% of unique human intron-exon boundaries, including 23 of 36 TG-AG splice site tandems), 100 nt flanking intron sequences were aligned using CLUSTALW [52], using the optimized parameter -gapopen = 2.2. The degree of conservation was determined for 50 nt of the human intron sequence upstream of the splice site (tandem), giving a score of 1 for an identical aligned nucleotide in mouse, a score of 0 for a mismatch, and a penalty of -1 for inserted mouse sequence. Since a histogram of sequence conservation in canonical introns showed a non-normal distribution, statistical testing was performed using a permutation test. Intron samples of given size were simulated by random drawings from the intron data set, and the average sequence identity was calculated, repeating the sampling procedure 10,000 times.

Where automated processing failed (13 of 36 TG-AG splice site tandems), orthologous intron-exon boundaries were retrieved using the UCSC genome browser. These cases were not used for the statistical analysis since these represent a likely biased subset with regard to sequence conservation, and an appropriate large data set for comparison is not available.

## Additional data files

The following additional data are available with the online version of this paper. Additional data file 1 is a Word file containing a description of the estimation of the amount of *cis*-regulatory sequence context, three supplementary tables and two supplementary figures. Supplementary Table 1 lists the putative unusual splice sites evident from EST-to-genome alignments that failed the quality checks. Supplementary Table 2 provides data about the comprehensive analysis of putative 3' TG splice sites suggested by spliced alignments of RefSeq transcripts. Supplementary Table 3 contains all primer sequences. Supplementary Figure S1 shows the distance-dependent occurrence of TG-AG and AG-AG splice acceptor tandems. Supplementary Figure S2 shows a LOGO representation of the TG 3' splice site sequence context.

## Supplementary Material

Additional data file 1 Supplementary Table 1 lists the putative unusual splice sites evident from EST-to-genome alignments that failed the quality checks. Supplementary Table 2 provides data about the comprehensive analysis of putative 3' TG splice sites suggested by spliced alignments of RefSeq transcripts. Supplementary Table 3 contains all primer sequences. Supplementary Figure S1 shows the distance-dependent occurrence of TG-AG and AG-AG splice acceptor tandems. Supplementary Figure S2 shows a LOGO representation of the TG 3' splice site sequence context. Click here for file
