# Supplementary material for: Violating the splicing rules: TG dinucleotides function as alternative 3' splice sites in U2-dependent introns
Source: Genome Biol. 2007 Aug 1;8(8):R154. doi: 10.1186/gb-2007-8-8-r154 (PMC2374985; doi:10.1186/gb-2007-8-8-r154)
Supplement: Additional data file 1 — Supplementary Table 1 lists the putative unusual splice sites evident from EST-to-genome alignments that failed the quality checks. Supplementary Table 2 provides data about the comprehensive analysis of putative 3' TG splice sites suggested by spliced alignments of RefSeq transcripts. Supplementary Table 3 contains all primer sequences. Supplementary Figure S1 shows the distance-dependent occurrence of TG-AG and AG-AG splice acceptor tandems. Supplementary Figure S2 shows a LOGO representation of the TG 3' splice site sequence context. [file gb-2007-8-8-r154-S1.doc]

1. **Violating the splicing rules: TG dinucleotides function as alternative 3' splice sites in U2-dependent introns**
2. **- Supplemental Material -**
3. Karol Szafranski, Stefanie Schindler, Stefan Taudien, Michael Hiller, Klaus Huse, Niels Jahn, Stefan Schreiber, Rolf Backofen, Matthias Platzer

# Index

[Index 1](#__RefHeading___Toc161055412)

[Estimating the amount of *cis*-regulatory sequence context 1](#__RefHeading___Toc161055413)

[Supplementary Tables 2](#__RefHeading___Toc161055414)

[Supplementary Figures 9](#__RefHeading___Toc161055415)

# Estimating the amount of *cis*-regulatory sequence context

1. The number of TGs functioning as splice acceptors is extremely small compared to the number of TG-AG tandems found at human intron-exon boundaries. For example, NTGNAG/NAGNTG motifs occur 58,374 times at intron-exon boundaries, whereas the TG is used as a splice site in 8 of those tandems (0.01%). For comparison, 8308 intron 3' ends display a NAGNAG tandem motif, and about 860 of these are alternatively spliced (10%). Given this tiny fraction of spliced 3' TGs in TG-AG tandems, *cis*-regulatory elements must play a crucial role in the definition of TG splice acceptors. The amount of required contextual sequence information can be estimated from the case numbers: assuming that spliced TGs evolve by chance, and estimating that about half of the cases do not underlie purifying selection (main document, fig. 3), then the empirical likelihood of evolving the necessary sequence context for splicing is 4/58374 ≈ 1·10^-4^. This corresponds to 13 bits, or 6.5 nucleotides, of sequence information. Given that regulatory sequence motifs are typically degenerate, the extent of constrained sequence context is certainly severalfold.

# Supplementary Tables

1. Supplement table 1. Putative unusual splice sites evident from EST-to-genome alignments that failed the quality checks.

|  | 1. **intron** | |  |  | 1. **ESTs for unusual 3'SS** | |  |
| --- | --- | --- | --- | --- | --- | --- | --- |
| 1. **gene** | 1. **#** | 1. **length** | 1. **distance** | 1. **3'SS motif** | 1. **frequency** | 1. **#** | 1. **comment** |
| 1. **alignment artifacts** |  |  |  |  |  |  |  |
| 1. *TSPAN1* | 1. 3 | 1. 196 | 1. 3 | 1. CAG|TTC, | 1. 0.495 | 1. 165 | false sequence/alignment for repetitive exon sequence |
| 1. *TTC1* | 1. 1 | 1. 1306 | 1. 3 | 1. CAG|CTT, | 1. 0.089 | 1. 15 | chronic mismatch of EST sequences; donor GTNGTN provokes the observed GTT indel |
| 1. **EST artifacts—AAA indels** |  |  |  |  |  |  |  |
| 1. *MACF1* | 1. 20 | 1. 3281 | 1. 3 | 1. CAG|TTT, | 1. 0.167 | 1. 1 |  |
| 1. *ASGR1* | 1. 7 | 1. 70 | 1. 3 | 1. CAG|AAA, | 1. 0.125 | 1. 1 |  |
| 1. *ITIH3* | 1. 1 | 1. 706 | 1. 3 | 1. CAG|AAA, | 1. 0.054 | 1. 3 | 1. rejected after EST re-sequencing |
| 1. *SCYL1* | 1. 3 | 1. 80 | 1. 3 | 1. CAG|AAA, | 1. 0.041 | 1. 3 | 1. rejected after EST re-sequencing |
| 1. *COPB* | 1. 4 | 1. 2962 | 1. 3 | 1. AAG|AAA, | 1. 0.034 | 1. 2 | 1. rejected after EST re-sequencing |
| 1. *ERAL1* | 1. 9 | 1. 1147 | 1. 3 | 1. CAG|AAA, | 1. 0.027 | 1. 2 | 1. rejected after EST re-sequencing |
| 1. *IFI30* | 1. 5 | 1. 418 | 1. 3 | 1. CAG|AAA, | 1. 0.003 | 1. 1 |  |
| 1. *SMAP* | 1. 2 | 1. 3412 | 1. 3 | 1. TAG|AAA, | 1. 0.002 | 1. 1 |  |
| 1. **EST artifacts—species** |  |  |  |  |  |  |  |
| 1. *SLC39A11* | 1. 1 | 1. 3835 | 1. 3 | 1. CAG|CTG, | 1. 0.036 | 1. 2 | 1. the two supporting ESTs (BG311118, CX752207) show mismatches to the human sequence but do perfectly match to the mouse RefSeq; in mouse, there's a NAGNAG tandem acceptor |

1. **Supplement table 2.** Comprehensive analysis of putative 3' TG splice sites suggested by spliced alignments of RefSeq transcripts. The “i-code” is an interpretation code for RefSeqs: +=valid, a=alignment artifact, i=lack of independent evidence, m=multiple splice variants, s=likely sequence artifacts, v=popul­ational variation mimicks TG splice site.

| 1. **gene** | 1. **TG RefSeq** | 1. **intron** | | 1. **3' SS** | 1. **TG splice** | | 1. **interpretation** | 1. **i-code** |
| --- | --- | --- | --- | --- | --- | --- | --- | --- |
|  |  | 1. **#** | 1. **length** |  | 1. **# mRNAs** | 1. **# ESTs** |  |  |
| 1. *DLG4* | 1. NM_001365 | 1. 5 | 1. 131 | 1. TCTGTCCCGTGCTG|GAGTTGCAGGTG |  | 1. 8 of 13 |  | 1. + |
| 1. *GNAS* | 1. NM_016592 | 1. 3 | 1. 4542 | 1. TTTCAATCCCACTG|CAGTGAGAAGGC |  |  |  | 1. + |
| 1. *PCBP2* | 1. NM_005016 | 1. 7 | 1. 1337 | 1. TTTTTTCCCCTCTG|ACTCTCTCCCAG | 1. 6 of 13 | 1. ~50% |  | 1. + |
| 1. *ARS2* | 1. NM_015908 | 1. 17 | 1. 182 | 1. CCCTGTCCGTGTTG|TACTCCCCCCAG | 1. 5 of 9 | 1. 80% |  | 1. + |
| 1. *RYK* | 1. NM_001005861 | 1. 7 | 1. 3098 | 1. GTTTGGCTTTGTTG|GCTCCTTAGGTT |  | 1. 80% |  | 1. + |
| 1. *LOC346653* | 1. NM_001012454 | 1. 1 | 1. 3097 | 1. TCTGCTCCTTTCTG|ACCCATGTACCT | 1. 2 of 2 | 1. 2 of 4 |  | 1. + |
| 1. *CACNA1A* | 1. NM_000068, NM_023035 | 1. 9 | 1. 2532 | 1. TGTTTCCATTGTTG|GAGCTCTGCGGA | 1. 5 of 6 | 1. 0 of 1 | 1. highly conserved; not a miniexon candidate | 1. + |
| 1. *SH3D19* | 1. NM_001009555 | 1. 6 | 1. 838 | 1. TTTTATTTGTTTTG|GTTTTGTTTTGG | 1. 1 of 2 (BX647422, clone DKFZ­p686I04144) | 1. 1 of 15 (BX405733, clone CS0­DM­008YI20) | 1. nice alignment; independent evidences; no conservation at all | 1. + |
| 1. *BAT3* | 1. NM_004639 | 1. 6 | 1. 832 | 1. CCTTTGGTATCCTG|ACTCTCCCCTAC | 1. 1 of 5 (M33519) | 1. 1 of ~130 (BI824648=ti:57196821) | 1. nice alignment; TG conserved until mouse/rat; M33519 -> Banerjee et al. 1990 | 1. + |
| 1. *CDH23* | 1. NM_022124, NM_052836 | 1. 11 | 1. 28431 | 1. CTTCTGCACTCTTG|ACCCAGGGCCTG | 1. 7 of 8 | 1. 0 of 4 | 1. 6-nt miniexon | 1. a(e) |
| 1. *BRP44L* | 1. NM_016098 | 1. 1 | 1. 15911 | 1. CCTCTCATTTTTTG|TAGCACTTCTGG | 1. 3 of 6 | 1. 1 of ~100 (AA401678) | 1. 4-nt miniexon | 1. a(e) |
| 1. *ASXL2* | 1. NM_018263 | 1. 2 | 1. 39140 | 1. TCTTCTTTGTTTTG|CAGTGGGACTTC |  | 1. 0 of 15 | 1. 3-nt miniexon, Katoh and Katoh 2002 | 1. a(e) |
| 1. *PITPNA* | 1. NM_006224 | 1. 4 | 1. 6526 | 1. AGTCAAGTTAACTG|TTATTACAAGGC | 1. 3 of 7 | 1. 1 of ~40 | 1. 8-nt miniexon, rare alignment variant | 1. a(e) |
| 1. *TNNT2* | 1. NM_001001430 | 1. 11 | 1. 1180 | 1. ACCTGGCCCTCCTG|CAGGCCTTGCTC | 1. ? of 12 | 1. 32 of 49 | 1. 6/9-nt miniexon | 1. a(e) |
| 1. *LOC440321* | 1. NM_001012452 | 1. 11 | 1. 3090 | 1. ACCTGAGTGAGCTG|GTGGAGAAAGAA | 1. 1 of 3 |  | 1. several paralogs | 1. a(r) |
| 1. *SIGLEC10* | 1. NM_033130 | 1. 11 | 1. 291 | 1. GCCTGGGCAACATG|GTGAAACCCCAT |  |  | 1. repetitive acceptor sequence | 1. a(r) |
| 1. *C5orf12* | 1. NM_178276 | 1. 11 | 1. 32455 | 1. TTCTCTTGCTGCTG|CCATGTAAGAAG |  |  | 1. repetitive acceptor sequence | 1. a(r) |
| 1. *PRR11* | 1. NM_018304 | 1. 10 | 1. 1888 | 1. GCCTGGCCAACATG|GTGAAATCCCAT |  |  | 1. repetitive acceptor sequence | 1. a(r) |
| 1. *SLC25A15* | 1. NM_014252 | 1. 7 | 1. 1198 | 1. ATTAGCTGGGCGTG|GTGGCACGTGCC | 1. 1 of 4 | 1. 0 of 11 | 1. repetitive acceptor sequence | 1. a(r) |
| 1. *F11R* | 1. NM_144502 | 1. 3 | 1. 302 | 1. TGCCTCCTCTTGTG|GTAGCTTCCTAT | 1. 1 of 15 | 1. 0 of ~500 |  | 1. i |
| 1. *LOC389607* | 1. NM_001013651 | 1. 1 | 1. 138 | 1. CCCTCCCCAGGATG|CTCAGTGCACAC | 1. 1 of 3 | 1. 0 of 2 |  | 1. i |
| 1. *PCDH17* | 1. NM_014459 | 1. 2 | 1. 126 | 1. TTTTTCTTTATATG|TATTTCAGTAGC | 1. 1 of 2 | 1. 0 of 4; ti:142957602: no TG splice | 1. nice alignment | 1. i |
| 1. *BCL11A* | 1. NM_138553 | 1. 4 | 1. 7997 | 1. TTCCCCCTCCTCTG|TCTCCAACCTCT | 1. 1 of 4 | 1. 0 of 13 |  | 1. i |
| 1. *MID1* | 1. NM_033291 | 1. 9 | 1. 5253 | 1. ACAATAACTGGGTG|GTGAGACACAAT | 1. 1 of 18 | 1. 0 of 7 | 1. splice contains premature stop in last exon | 1. i |
| 1. *AIM1L* | 1. NM_017977 | 1. 2 | 1. 22989 | 1. CAGGCTCCAAGGTG|GTGCTGTGGGCC | 1. 1 of 2 (AK000902, re-sequenced clone HEMBA­1001009) | 1. 1 of 10 (AU144147, clone HEMBA­1001009) | 1. intron in 3UTR | 1. i |
| 1. *UBE3B* | 1. NM_183414 | 1. 25 | 1. 3466 | 1. TCTCTTCCTTGTTG|GCAACAGAATTA | 1. 1 of 7 (AL096740) | 1. 0 of 50 |  | 1. i |
| 1. *MPDZ* | 1. NM_003829 | 1. 39 | 1. 1310 | 1. TTTTCCACTCTCTG|GATCCAGTACAT | 1. 1 of 9 | 1. 0 of 19 |  | 1. i |
| 1. *RAD51* | 1. NM_133487 | 1. 3 | 1. 17684 | 1. CAGAACGGCTGCTG|GCAGTGGCTGAG | 1. 1 of 10 (BC001459, fully sequenced EST BE280848=IMAGE:3139011) | 1. 1 of 45 (BE280848) |  | 1. i |
| 1. *STARD7* | 1. NM_139267 | 1. 1 | 1. 215 | 1. GCCCCTCCGGACTG|GTTCCTTGGGCC | 1. 2 of 12 | 1. 3 of 35; all derived from NIH_MGC_19 (neuro­blast­oma) | 1. mix of different splices: retention + 2 overlapping introns; PPT missing; no conservation | 1. m |
| 1. *FLJ31846* | 1. NM_144974 | 1. 8 | 1. 4905 | 1. TTAAGATTCTTTTG|AACTTTTTCATT | 1. 1 of 2 | 1. 0 of 2 | 1. weird splice: 4 transcripts, 3 intron variants; intron in 3UTR | 1. m |
| 1. *SPRED1* | 1. NM_152594 | 1. 1 | 1. 766 | 1. CCTCGGTGCTGCTG|TTGCTCCCCCGC | 1. 2 of 4 | 1. 1 of 6 | 1. 3 different splices (intron variants) found in 8 ESTs -> cloning artifact for G+C-rich sequence? | 1. m |
| 1. *YARS2* | 1. NM_015936 | 1. 5 | 1. 2550 | 1. AATCTTAGAGCCTG|GTGTAAGTGCTC | 1. 1 of 12 (AF132939) | 1. 0 of 2 | 1. atypical mRNA AF132939: 3'UTR overlapping with 3'UTR from neighboring gene | 1. m |
| 1. *HDC* | 1. NM_002112 | 1. 4 | 1. 2757 | 1. ATCCTTTTCCCCTG|CAGAGCACGGTC | 1. 1 of 2 (X54297) | 1. 0 of 8 | 1. founder of RefSeq, mRNA X54297, shows mismatching alignment at splice site | 1. s |
| 1. *PERQ* | 1. NM_022574 | 1. 8 | 1. 145 | 1. TTCTTCTTTTTCTG|GGCATCCAGGAG | 1. 0 of 1 | 1. 0 of 6 | 1. RefSeq surprisingly based on BAC sequence AF053356 | 1. s |
| 1. *GANAB* | 1. NM_198335 | 1. 18 | 1. 1424 | 1. CCTAGGCCCCTGTG|GGTGCAGTACCC | 1. 0 of 9 | 1. 0 of ~100 | 1. RefSeq shows deletion versus all mRNAs, including the founders; corrected by NCBI 2006-02-28 | 1. s |
| 1. *ASB15* | 1. NM_080928 | 1. 9 | 1. 686 | 1. TGTTCCTCTGTGTG|CTAAACTGAAGT | 1. 1 of 4 (AF428257) | 1. 0 of 1 | 1. NCBI; RefSeq + founder AF428257 show two major indels | 1. s |
| 1. *BAZ2A* | 1. NM_013449 | 1. 1 | 1. 18654 | 1. CTCCTGCAGAAATG|GAGGCAAACGAC | 1. 1 of 3 (AB032254) | 1. 0 of 7 | 1. serious errors (indels) in mRNA entry AB032254 | 1. s+a |
| 1. *CEACAM21* | 1. NM_033543 | 1. 6 | 1. 592 | 1. CTCTGTTTTTACTG|GAATTGCTACAC | 1. 4 of 5 | 1. 4 of 7 | 1. SNP rs3745936 | 1. v |
| 1. *NPHP4* | 1. NM_015102 | 1. 20 | 1. 1992 | 1. CCTCTTGTCTGCTG|GCGCAGCAGAGC | 1. 2 of 2 | 1. 5 of 6 | 1. SNP rs1287637 | 1. v |

1. **Supplementary t**able 3. Listing of primer sequences. All sequences are given in 5'-3' orientation.

ARS2.i17, RT-PCR of splice junction

nesting step A

ARS2.i17.u1 GCAGAGAAAATTGAGGAAGTG

ARS2.i17.d1 CCTCGGAAGGCATCATAG

nesting step B

ARS2.i17.u2 TAACAACTTCCTCACTGATGC

ARS2.i17.d2 CTCGACCAGCACCATAC

ARS2.i17, PCR of intron

same primer set as for RT-PCR

ARS2.i17, pyrosequencing of splice junction

ARS2.i17.upy CACCTGGCCCCGC

ARS2.i17.dpy GTCCTGGGGTCAAAC

BRUNOL4.i6, RT-PCR of splice junction

nesting step A

BRUNOL4.i6.u1 CAGTCTGGTGGTCAAGTTC

BRUNOL4.i6.d1 GGTCATAGGTGCGGC

nesting step B

BRUNOL4.i6.u2 CACGATGCGGCGAATG

BRUNOL4.i6.d2 CGCCATCTGCTGCATCT

BRUNOL4.i6, PCR of intron

nesting step A

BRUNOL4.i6.ug1 CTGTTGAGGTACAGGGCA

BRUNOL4.i6.d1

nesting step B

BRUNOL4.i6.ug2 CTTTCTGGAGTGGTAGTTGG

BRUNOL4.i6.d2

CACNA1A.i9, RT-PCR of splice junction

nesting step A

CACNA1A.i9.u1 TGGAACTGGTTGTACTTCATC

CACNA1A.i9.d1 GAACAATAGCAACACACAGC

nesting step B

CACNA1A.i9.u2 TTTTTATGCTGAACCTTGTG
 CACNA1A.i9.d2 CTTGGCACTTTTAATGCTG

c21orf63.i3, RT-PCR of splice junction

nesting step A

C21orf63.i3.u1 GCTGGACGAATGCCAGAAC

C21orf63.i3.d1 CCTTGGAGGAGCAGATGTC

nesting step B

C21orf63.i3.u2 GCCACCTCCTGGTCAATAG

C21orf63.i3.d2 GTCGCAGAGTAGATGTTGAGG

c21orf63.i3, PCR of intron

nesting step A

C21orf63.i3.ug1 GTTTAATTCCCAGGGTCTTTGC

C21orf63.i3.d1

nesting step B

C21orf63.i3.ug2 TACTTCCCACCCGCTTCC

C21orf63.i3.d2

FBXO17.i3, RT-PCR of splice junction

nesting step A

FBXO17.i3.u1 CCATAGAAAAGAACCTAACACC

FBXO17.i3.d1 CTGACTGTGACTTGAATGCC

nesting step B

FBXO17.i3.u2 GGCTCCTTCGCAGAC

FBXO17.i3.d2 CCTTCCATCACCAGGTC

FBXO17.i3, PCR of intron

nesting step A

FBXO17.i3.ug1 CAGGTCTGCTAGAGCAAAC

FBXO17.i3.d1

nesting step B

FBXO17.i3.ug1 GACTTCTTGGAGGAGGTG

FBXO17.i3.d2

GNAS.i3, RT-PCR of splice junction

nesting step A

GNAS.i3.u1 GTTTAATGGAGAGGGCGGC

GNAS.i3.d1 GTCAAAGTCAGGCACGTTCA

nesting step B

GNAS.i3.u2 CAGGCTGCAAGGAGCAAC

GNAS.i3.d1

LOC346653.i1, RT-PCR of splice junction

nesting step A

LOC346653.i1.u1 AAGCCCATTCTTACCAAGAACC

LOC346653.i1.d1 GTCGTAGATTCGTAGCTCCAC

nesting step B

LOC346653.i1.u2 CTTTGTCAACTGATTCATTCTCC

LOC346653.i1.d2 CTGCTGAGGTTGATGACTG

LOC346653.i1, PCR of intron

nesting step A

LOC346653.i1.ug1 CCCAGAGAAGAATGACACAG

LOC346653.i1.d1b CTGCTGAGGTTGATGACTG

nesting step B

LOC346653.i1.ug2 TGTATTCCACCCCTTGTTTTC

LOC346653.i1.d2b CAGACCCTTCACAGACATC

LOC55795.i2, RT-PCR of splice junction

nesting step A

LOC55795.i2.u1 GAAGCCATCGACAGCAG

LOC55795.i2.d1 GCTGCAAACATTTCATCATAAGG

nesting step B

LOC55795.i2.u2 GATGGAGCATCTTGTGCAG

LOC55795.i2.d2 AAGACTTGTTGACACTTCTCC

LOC55795.i2, PCR of intron

nesting step A

LOC55795.i2.ug1 GCATAAATTCTCTTGAAACAGAGG

LOC55795.i2.d1

nesting step B

LOC55795.i2.ug2 ATGTTGCATTCTTTCTGTGCC

LOC55795.i2.d2

PCBP2.i7, RT-PCR of splice junction

nesting step A

PCBP2.i7.u1 GGATATGCTACCCAACTCAAC

PCBP2.i7.d1 GACCACCTGCAAAGATGAC

nesting step B

PCBP2.i7.u2 CACTATTGCTGGCATTCCAC

PCBP2.i7.d2 GAGCTGGACGGCTTG

PCBP2.i7, PCR of intron

nesting step A

PCBP2.i7.ug1 GAGACAATTTGGTAGGTAAGG

PCBP2.i7.d1

nesting step B

PCBP2.i7.ug2 CCTGTGTGAGCTAAAGCC

PCBP2.i7.d2

PCGF2.i1, RT-PCR of splice junction

nesting step A

PCGF2.i1.u1 GCGAGCGACACGGCTG

PCGF2.i1.d1 CTCGGAACAGGGTCTGC

nesting step B

PCGF2.i1.u2 GGACCCCGAACCCAG

PCGF2.i1.d2 GGAGACGCCAAATCGTTAAG

PCGF2.i1, PCR of intron

same primer set as for RT-PCR

TNNT2.i1, RT-PCR of splice junction

nesting step A

TNNT2.i1.u1 CGCTGAGACTGAGCAGAC

TNNT2.i1.d1 CTCTGCTTCAGCATCCTCTTC

nesting step B

TNNT2.i1.u2 ACGCCTCCAGGATCTGTC

TNNT2.i1.d2 ATCCTCTTCCGCTGCCTC

TNNT2.i1, PCR of intron

nesting step A

TNNT2.i1.ug1 GCAAGGAACGAAGTGGACATC

TNNT2.i1.dg1 GGAAATGGCTATATCTCTCCTC

nesting step B

TNNT2.i1.ug2 CCATGTGGGTGTCACTATCTC

TNNT2.i1.dg2 CAGCTACTTCTACCCAGAATCC

ZNF9.i3, RT-PCR of splice junction

nesting step A

ZNF9.i3.u1 TCGCTGTGGTGAGTCTG

ZNF9.i3.d1 GCTCTCGCTCTCTCTTG

nesting step B

ZNF9.i3.u2 GGTGAGTCTGGTCATCTTG

ZNF9.i3.d2 GCAGTCCTTGGCAATGTG

ZNF9.i3, PCR of intron

same primer set as for RT-PCR

ZNF9.i3, pyrosequencing splice junction

ZNF9.i3.upy GATCTTCAGGAGGAT

ZNF9.i3.dpy CCGCAGTTATAGCA

# Supplementary Figures

1. **Supplementary figure S1.** Distance-dependent occurrence of TG‑AG and AG‑AG splice acceptor tandems. The histogram bars for the TG‑AG tandems are proportionally stretched (factor 8x) for better comparability.

1. **Supplementary figure S2.** LOGO representation of the sequence context of TG 3’ splice sites. The image was produced from 38 aligned *bona fide* splice sites obtained from 36 introns using a modified makelogo program from T. Schneider (http:// www.ccmp.ncifcrf.gov/ ~toms/ logoprograms.html). The total height of symbol stacks at each alignment position displays the information content scaled to bits.
